# Interactions between inflammatory gene polymorphisms and HTLV-I infection for total death, incidence of cancer, and atherosclerosis-related diseases among the Japanese population

**DOI:** 10.1016/j.je.2016.08.017

**Published:** 2017-05-30

**Authors:** Tara Sefanya Kairupan, Rie Ibusuki, Motahare Kheradmand, Yasuko Sagara, Eva Mariane Mantjoro, Yora Nindita, Hideshi Niimura, Kazuyo Kuwabara, Shin Ogawa, Noriko Tsumematsu-Nakahata, Yasuhito Nerome, Tetsuhiro Owaki, Toshifumi Matsushita, Shigeho Maenohara, Kazunari Yamaguchi, Toshiro Takezaki

**Affiliations:** a Department of International Islands and Community Medicine, Kagoshima University Graduate School of Medical and Dental Sciences, Kagoshima, Japan; b Faculty of Medicine, Sam Ratulangi University, Manado, Indonesia; c Mazandaran University of Medical Sciences, Sari, Iran; d Japanese Red Cross Kyushu Block Blood Center, Fukuoka, Japan; e Faculty of Public Health, Sam Ratulangi University, Manado, Indonesia; f Faculty of Medicine, Diponegoro University, Semarang, Indonesia; g Yonemori Hospital, Kagoshima, Japan; h Department of Preventive Medicine and Public Health, Keio University School of Medicine, Tokyo, Japan; i National Health Insurance Yamato Clinic, Yamato, Japan; j Department of Community Medicine Management, Faculty of Medicine, Shimane University, Izumo, Japan; k Education Center for Doctors in Remote Islands and Rural Areas, Kagoshima University Graduate School of Medical and Dental Sciences, Kagoshima, Japan; l Japan Health Club, Kagoshima, Japan; m JA Kagoshima Kouseiren Medical Health Care Center, Kagoshima, Japan; n National Institute of Infectious Diseases, Tokyo, Japan

**Keywords:** HTLV-I, Gene polymorphism, Inflammation, Interaction

## Abstract

**Background:**

An increased risk of total death owing to human T-lymphotropic virus type-I (HTLV-I) infection has been reported. However, its etiology and protective factors are unclear. Various studies reported fluctuations in immune-inflammatory status among HTLV-I carriers. We conducted a matched cohort study among the general population in an HTLV-I-endemic region of Japan to investigate the interaction between inflammatory gene polymorphisms and HTLV-I infection for total death, incidence of cancer, and atherosclerosis-related diseases.

**Method:**

We selected 2180 sub-cohort subjects aged 35–69 years from the cohort population, after matching for age, sex, and region with HTLV-I seropositives. They were followed up for a maximum of 10 years. Inflammatory gene polymorphisms were selected from *TNF-*α, *IL-10,* and *NF-*κ*B1*. A Cox proportional hazard model was used to estimate the hazard ratio (HR) and the interaction between gene polymorphisms and HTLV-I for risk of total death and incidence of cancer and atherosclerosis-related diseases.

**Results:**

HTLV-I seropositivity rate was 6.4% in the cohort population. The interaction between *TNF*-α 1031T/C and HTLV-I for atherosclerosis-related disease incidence was statistically significant (*p* = 0.020). No significant interaction was observed between *IL-10* 819T/C or *NF-*κ*B1* 94ATTG ins/del and HTLV-I. An increased HR for total death was observed in the Amami island region, after adjustment of various factors with gene polymorphisms (HR 3.03; 95% confidence interval, 1.18–7.77).

**Conclusion:**

The present study found the interaction between *TNF*-α 1031T/C and HTLV-I to be a risk factor for atherosclerosis-related disease. Further follow-up is warranted to investigate protective factors against developing diseases among susceptible HTLV-I carriers.

## Highlights

• We observed death and incidence risk with HTLV-I and SNPs in a matched cohort study.
• An increased risk for total death with HTLV-I was observed in the island region.
• Inflammatory SNP interacted with HTLV-I for atherosclerosis-related disease risk.

## Introduction

Human T-lymphotropic virus type-I (HTLV-I) is an oncogenic human retrovirus infecting approximately 15–25 million people worldwide.^1^ There are approximately 1.08 million carriers in Japan, mostly distributed in the Kyushu district, in the southern part of Japan.^2^ While the majority of the infected individuals remain asymptomatic throughout their lives, up to 5% of carriers develop adult T-cell leukemia/lymphoma (ATLL), HTLV-I associated myelopathy/tropical spastic paraparesis (HAM*/*TSP),^2^ and other HTLV-I related diseases, such as uveitis,^3^ pneumonitis,^4^ and arthritis.^5^ ATLL is the most serious disease, with a 4-year overall survival rate of 11% for the acute type.^6^ On the contrary, its effect on total deaths is relatively lower, attributing to 0.08% of total deaths and 0.3% of all types of cancer in 2014 in Japan.^7^ However, several prospective studies reported an increased risk of mortality for causes other than ATLL and non-neoplastic diseases,^8^, ^9^, ^10^ and another study noted the frequency of reported history of asthma and cardiac disease among those with HTLV-I infection.^11^ Furthermore, a cross-sectional study revealed that HTLV-I infection was a risk factor for atherosclerosis, as evaluated by carotid intima-media thickness (IMT).^12^ However, its etiology and protective factors are unclear.

Fluctuation in the immune-inflammatory status among HTLV-I carriers has been reported in many studies. Increased production of T-helper (Th)-1 pro-inflammatory cytokines (IFN-*γ*, TNF-*α*, and IL-2), along with a reduction of T-regulatory and Th-2 cytokines (IL-10 and IL-4), was observed in HTLV-I-associated inflammatory diseases, due to Tax induced signaling abnormalities and persistent NF-*κ*B1 activation of the infected CD4+ CD25+ CCR+ T-cells.^13^, ^14^ Interestingly, these common cytokines are also involved in the development of atherosclerosis^15^ and cancers.^16^

Therefore, it is reasonable that immune-inflammatory processes associated with persistent HTLV-I infection may be involved in modifying the risk of various disease outcomes. It also leads to the question of whether individual susceptibility to the immune-inflammatory mechanism may modify the disease risk in HTLV-I infection. However, no study has used inflammatory gene polymorphisms to investigate the association between the risk and individual susceptibility to the immune-inflammatory mechanism. To investigate the interaction between inflammatory gene polymorphisms and HTLV-I infection for total death and the incidence of cancer and atherosclerosis-related diseases, we conducted a matched cohort study among the general population in a HTLV-I-endemic region of Japan.

## Methods

### Study subjects

**Fig. 1:**
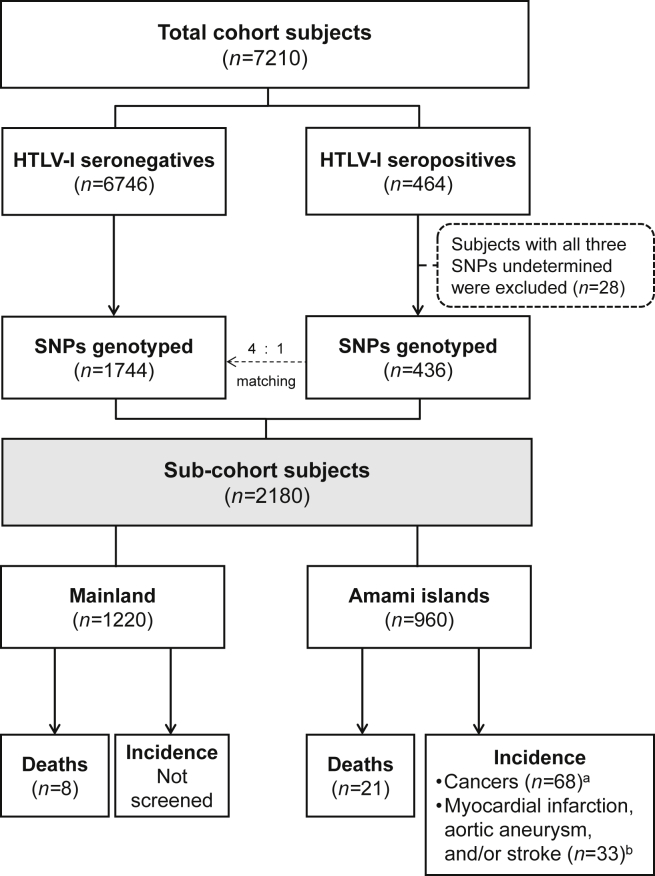
Flow-chart of the selection process of the sub-cohort subjects. ^a^Included 5 death cases. ^b^Included 1 death case.

Study subjects were recruited from the general population of Kagoshima Prefecture of Kyushu district, Japan. This study was conducted as a part of the Japan Multi-institutional Collaborative Cohort (J-MICC) study, which has been described elsewhere.^17^, ^18^, ^19^ We conducted baseline surveys in five Amami island regions from 2005 to 2008 and three mainland regions in 2012. The subjects were individuals undergoing health-checkups that were conducted by the local government or private companies, and those aged 32–78 years were asked to participate in the study by filling informed consents (response rate: 69.8%). The baseline surveys consisted of a questionnaire survey, blood collection, and examination for arterial stiffness using the cardio-ankle vascular index (CAVI). We included subjects with complete data and blood samples and excluded subjects with measurement error conditions of CAVI.^18^ Additionally, subjects with indeterminate results of HTLV-I seropositivity and withdrawals from the study during the follow-up were excluded. The number of eligible subjects at the baseline was 7210 (2956 men and 4254 women). HTLV-I seropositivity for all eligible subjects was examined. We selected all HTLV-I seropositive subjects and four-time seronegative subjects, after matching for age, sex, and the regions of island or mainland, with each seropositive subject as sub-cohort subject, to examine gene polymorphisms (Fig. 1). We adopted this strategy because 1) the distribution of HTLV-I seropositive subjects was older than the seronegatives and matching is more appropriate than adjusting to compare the effect of inflammatory gene polymorphisms, in terms of similar immunological background by age; 2) a nested case–control study design requires complicated control setting for three different outcomes of death and the incidence of cancer and atherosclerosis-related diseases; 3) the number of HTLV-I seropositive subjects was limited and the larger number of seronegative subjects contributed to a relatively small increase in statistical power. At first, seronegative subjects were selected according to the same age, sex, and town/city of each seropositive subject. When the number of complete matched seronegative subjects was limited, the following rules were applied in order: 1) neighboring town/city in each Amami and mainland region, respectively; 2) +1 year of age; 3) −1 year of age; 4) adding age for candidates. Ten seronegative subjects were selected from other region, after matching for age and sex, because of the lack of matched candidates. Twenty-eight HTLV-I seropositive subjects were excluded from the sub-cohort study owing to indeterminate gene polymorphism results. The final eligible subjects of the sub-cohort comprised 2180 individuals aged 35–69 years (436 seropositives and 1744 seronegatives). The present study was approved by the ethics review committee for human genome/gene analysis research at our institution.

We followed up with all subjects to account for movement out of the study region or death using local government records until the end of 2015, with the exception of subjects from one mainland region, who were followed up until 2013. This follow-up in the total cohort revealed 238 cases of movement out of the study region and 164 deaths. There were 29 deaths in the sub-cohort subjects.

Information regarding the incidence of cancer, myocardial infarction, aortic aneurysm, and stroke was collected using direct interview at 5–6 and 10 years after the baseline. For subjects whom we were unable to interview, information was collected via mail. The responses to the question on incidence in both the interview and the questionnaire always included “yes”, as well as “not suffered”. Causes of death were collected using death certificate records from the public health center, and deceased subjects were included in incident cases. The information of incident cases was not yet obtained for the mainland region, because the baseline survey was conducted in 2012 and the follow-up survey for incident cases of the mainland is scheduled in 2017. The coverage rate of the incident survey was 81.3% in the Amami island regions. The numbers of incident cases of cancer, myocardial infarction, aortic aneurysm, and stroke were 68, 17, 2, and 14, respectively. The survey to the medical clinics/hospitals to confirm the diagnosis for screened cases is currently ongoing. We partially evaluated the positive predictive value for confirmed diagnosis in screened incident cases of cancer and atherosclerosis-related disease among those whom we could survey by May, 2016. The number of confirmed cases using medical records in the medical clinics/hospitals was 22 of 23 screened cases (95.6%) for cancer incidence and 15 of 16 (93.8%) screened cases for atherosclerosis-related disease incidence.

### Data collection

We used a J-MICC standardized structured questionnaire to collect individual information about socio-demographic characteristics, smoking, alcohol consumption, exercise, dietary habits, personal and family medical history, intake of prescription medicines and supplements, reproductive history, and stress status.^17^

Health checkup data obtained from the Kagoshima Kouseiren Health Center and Japan Health Club, included systolic and diastolic blood pressure, levels of total cholesterol, triglycerides (TG), high-density lipoprotein cholesterol (HDL-C), low-density lipoprotein cholesterol (LDL-C), and fasting blood glucose (FBG). The subjects were asked to fast for >10 h. LDL-C levels were calculated according to the Friedewald formula using a TG level <400 mg/dL^20^ when LDL-C examination was not included in the routine health checkup.

CAVI using Vasera VS-1000 and VS-1500 vascular screening systems (Fukuda Denshi Co., Ltd., Tokyo, Japan) was used to evaluate arterial stiffness.^21^, ^22^

Plasma samples were obtained at the baseline and were stored at −80 °C prior to the examination. We screened for HTLV-I antibody using a particle agglutination assay and confirmed using western blot analysis at the laboratory of the Fukuoka Red Cross Center and SRL Inc. Subjects with indeterminate western blot results were excluded from the study.

### DNA extraction and genotyping single-nucleotide polymorphisms

DNA was extracted from the buffy coat fraction using the standard method and the QIAamp Blood Mini Kit (Qiagen, Valencia, CA, USA), GenElute Blood Genomic DNA Kit (Sigma–Aldrich, St. Louis, MO, USA), or Blood-Animal-Plant DNA Preparation Kit (Jena Bioscience, Jena, Germany).

We selected three single-nucleotide polymorphisms (SNPs) with minor allele frequencies (MAF) greater than 0.05 from the common genes for cytokines that have important roles in the immune-inflammatory processes of HTLV-I infection, cancers, and atherosclerosis, including *TNF*-α 1031T/C [rs1799964], *IL-10* 819T/C [rs1800871], and *NF-*κ*B1* 94ATTG ins/del [rs28362491].^23^, ^24^, ^25^, ^26^, ^27^, ^28^, ^29^, ^30^, ^31^, ^32^, ^33^, ^34^, ^35^, ^36^, ^37^ These SNPs were genotyped using TaqMan allelic discrimination kits (Applied Biosystems, Foster City, CA, USA) and real-time polymerase chain reaction (StepOne, Applied Biosystems). Each reaction was performed in a 10 μL volume that contained 1 μL of genomic DNA (10 mg/μL), 5 μL of TaqMan Universal PCR Master Mix II, 0.125 μL of 40 × genotyping assay mix, and 3.875 μL of dH_2_O. The cycling was initiated by heating at 95 °C for 10 min, followed by 50 cycles each at 95 °C for 15 s, 58 °C for 1 min, and 25 °C for 30 s.

### Statistical analysis

Hypertension was defined based on a systolic blood pressure ≥140 mm Hg, diastolic blood pressure ≥90 mm Hg, or treatment with antihypertensive medication. Dyslipidemia was defined based on TG levels ≥150 mm/dL, LDL-C levels ≥140 mg/dL, HDL-C levels <40 mg/dL, or the use of antihyperlipidemic medication. Glucose intolerance was defined as FBG value ≥110 mg or those who were being administered diabetes mellitus treatment. Smokers were defined as current smokers, and ≥20 pack-years. Alcohol consumers were defined as those who currently consumed alcohol three times or more per week. Selected food and beverage intake was categorized according to an intake frequency of three times or more per week. Metabolic equivalents (METs) were calculated based on the frequency, intensity, and duration of habitual exercise and daily activities (walking and hard work), as obtained from the questionnaire. Estimation of METs per day was based on duration and the intensity of the exercise, using values of 3.0 for walking, 3.3 for light exercise, 4.0 for moderate exercise, 4.5 for heavy work, and 8.0 for heavy exercise.^38^ Arterial stiffness was defined as a CAVI value ≥ 9.0.^39^ We assigned dummy variables (0 and 1) for the genotypes of *TNF*-α 1031T/C ([CC & CT] and TT), *IL-10* 819T/C ([CC & CT] and TT), and *NF-*κ*B1* 94ATTG (ins/ins and [ins/del & del/del]).

The participants' background characteristics, atherosclerosis-related factors, current and past medical history, and the genotype distribution of SNPs were compared according to HTLV-I seropositivity by sex, and differences in the prevalence were evaluated using the chi-square test. The differences in genotype distribution from the Hardy–Weinberg equilibrium (HWE) were evaluated using Pearson's chi-square test.

Person-years were calculated from the date of the baseline until the date of death, movement out of the study area, incidence of cancer and atherosclerosis-related diseases, or the end of the follow-up, whichever came first. The date of the end of the follow-up was set as December 31, 2015 for death and movement out of the study area, except for one mainland region, for which it was set as December 31, 2013, and the most recent surveyed date was used for incidence. A Cox proportional hazard model was used to estimate the hazard ratios (HRs) and its 95% confidential intervals (CIs) for death and incidence with HTLV-I positivity, after adjustments for smoking, drinking, meat intake, hypertension, dyslipidemia, glucose intolerance, family history of coronary heart disease (CHD) and stroke, overweight, and exercise habit. The incident cases of myocardial infarction, aortic aneurysm, and stroke were combined as atherosclerosis-related diseases, because of the limited numbers of events. The interaction between each gene polymorphism and HTLV-I for the HR of death and incidence with HTLV-I was tested, after adjustment for related factors. All statistical analyses were performed using Stata software (version 12; Stata Corp., Collage Station, TX, USA), and differences with a *p*-value of <0.05 were considered statistically significant.

## Results

The mean age of the total cohort subjects was 57.3 (standard deviation [SD], 8.7; range, 32–69) years for men and 57.5 (SD, 8.1; range 34–78) years for women. The HTLV-I seropositivity rate was 6.4% (464/7210 subjects), with women showing a higher rate than men (7.0% vs. 5.6%). The seropositivity rate increased with age in both sexes (*p* < 0.001). The seropositivity rate differed by region, with mainland subjects of both sexes showing a higher rate than Amami island subjects (9.2% vs. 3.8% in men and 9.9% vs. 5.5% in women) (data not shown).

The prevalence of atherosclerosis-related factors and selected lifestyles was not different according to HTLV-I seropositivity for either men or women, except in relation to meat intake in women (Table 1).

**Table 1:** Clinical features and lifestyles of the sub-cohort study subjects at baseline according to HTLV-I seropositivity by sex.

	Number (%)					
	Men (n = 770)			Women (n = 1410)		
	HTLV-I (−)	HTLV-I (+)	*p-*value^a^	HTLV-I (−)	HTLV-I (+)	*p-*value^a^
Age, years						
35–49	52 (8.4)	13 (8.4)		72 (6.4)	18 (6.4)	
50–59	136 (22.1)	34 (22.1)		288 (25.5)	72 (25.5)	
60–69	428 (69.5)	107 (69.5)	1.000	768 (68.1)	192 (68.1)	1.000
Total	616 (100)	154 (100)		1128 (100)	282 (100)	
Region						
Amami islands	253 (41.1)	63 (40.9)		569 (50.4)	140 (49.7)	
Mainland	363 (58.9)	91 (59.1)	0.971	559 (49.6)	142 (50.3)	0.811
Prevalence of						
Hypertension	330 (53.5)	82 (53.3)	0.942	542 (48.1)	137 (48.6)	0.873
Dyslipidemia	303 (49.3)	78 (53.8)	0.327	606 (54.9)	134 (51.7)	0.359
Glucose intolerance	144 (23.6)	33 (22.0)	0.684	120 (10.8)	29 (11.2)	0.873
Family history of CHD and stroke	203 (38.2)	49 (36.8)	0.768	392 (40.0)	97 (41.0)	0.822
Overweight (BMI ≥25)	247 (40.1)	52 (34.7)	0.221	327 (29.3)	78 (30.0)	0.931
Arterial stiffness (CAVI ≥9.0)	203 (33.3)	58 (38.0)	0.286	201 (18.0)	51 (18.4)	0.887
Smoking (≥20 pack-years)	86 (18.5)	24 (21.0)	0.561	48 (4.8)	15 (6.0)	0.418
Alcohol drinking (≥3 times/week)	328 (53.8)	70 (45.8)	0.076	270 (24.0)	69 (24.6)	0.834
Meat intake (≥3 times/week)	185 (30.1)	43 (28.1)	0.632	481 (43.0)	96 (34.2)	0.007
Fish intake (≥3 times/week)	344 (56.0)	80 (52.3)	0.405	629 (56.2)	174 (62.1)	0.070
Green vegetables intake (≥3 times/week)	261 (42.6)	61 (39.9)	0.544	553 (49.3)	120 (42.9)	0.054
Green tea intake (≥3 times/week)	492 (80.3)	128 (83.7)	0.338	1000 (89.5)	257 (91.5)	0.336
Daily activities (≥15 METs/day)	338 (55.1)	93 (60.8)	0.201	593 (53.0)	146 (52.3)	0.842
Habitual exercises (≥0.73 METs/day)	305 (51.2)	76 (51.0)	0.971	580 (53.6)	141 (52.4)	0.738

BMI, body mass index; CAVI, cardio-ankle vascular index; CHD, coronary heart disease; METs, metabolic equivalents.

a Tested by chi-square test.

The genotype distribution of *TNF-*α 1031T/C, *IL-10* 819T/C, and *NF-*κ*B1* 94ATTG ins/del polymorphisms was not different between HTLV-I seronegatives and seropositives in either sex, except *NF-*κ*B1* 94ATTG ins/del polymorphism in women (Table 2).

**Table 2:** Distribution of genotypes of inflammatory gene polymorphisms according to HTLV-I seropositivity by sex in the sub-cohort study subjects.

	Number (%)					
	Men (n = 770)			Women (n = 1410)		
	HTLV-I (−)	HTLV-I (+)	*p-*value^a^	HTLV-I (−)	HTLV-I (+)	*p-*value^a^
*TNF-*α 1031T/C						
CC	20 (3.3)	6 (3.9)		46 (4.1)	13 (4.7)	
CT	176 (28.6)	52 (33.8)		341 (30.3)	91 (32.6)	
TT	418 (68.1)	96 (62.3)	0.400	740 (65.6)	175 (62.7)	0.644
*IL-10* 819T/C						
CC	77 (12.5)	18 (11.7)		172 (15.3)	51 (18.2)	
CT	294 (47.8)	69 (44.8)		534 (47.4)	120 (42.9)	
TT	244 (39.7)	67 (43.5)	0.687	421 (37.3)	109 (38.9)	0.306
*NF-*κ*B1* 94ATTG ins/del						
ins/ins	268 (43.7)	70 (45.4)		473 (42.0)	94 (33.6)	
ins/del	255 (41.5)	66 (42.9)		498 (44.2)	138 (49.3)	
del/del	91 (14.8)	18 (11.7)	0.608	156 (13.8)	48 (17.1)	0.032

a Tested by chi-square test.

The MAF of the *TNF-*α 1031T/C, *IL-10* 819T/C, and *NF-*κ*B1* 94ATTG ins/del polymorphisms were 0.186, 0.381, and 0.364, respectively, and the former two genotype frequencies were within the HWE (*p* = 0.383 and *p* = 0.735, respectively); however, the *NF-*κ*B1* 94ATTG ins/del polymorphism was not (*p* = 0.013). The MAF of the *TNF-*α 1031T/C (0.215 in Amami and 0.169 in the mainland, *p* < 0.001) and *IL-10* 819T/C (0.438 in Amami and 0.328 in the mainland, *p* < 0.001) were different by region, but that of the *NF-*κ*B1* 94ATTG ins/del was similar (0.363 and 0.365, respectively) (data not shown).

The HRs for death and incidence with HTLV-I were estimated by genotype group and each interaction was evaluated. The HRs of the CC & CT genotypes of *TNF*-α 1031T/C for total death and incidence of cancer and atherosclerosis-related diseases with HTLV-I seem to be higher than those of the TT genotype, and the interaction between *TNF*-α 1031T/C polymorphism and HTLV-I for incidence of atherosclerosis-related diseases was statistically significant (*p* = 0.020; Table 3). No significant interaction was observed between *IL-10* 819T/C and *NF-*κ*B1* 94ATTG ins/del polymorphisms and HTLV-I for total death and incidence rate of cancer or cardiovascular diseases.

**Table 3:** Hazard ratios for total deaths and incidence of cancer and atherosclerosis-related diseases with HTLV-I seropositivity according to inflammatory gene polymorphisms and their interactions in the sub-cohort study subjects.

	Death^a^		Incidence^b^			
	Total		Cancer		Atherosclerosis-related diseases^c^	
	Person-years (E/NE)	HR^d^ (95% CI)	Person-years (E/NE)	HR^d^ (95% CI)	Person-years (E/NE)	HR^d^ (95% CI)
Total						
HTLV-I (−)	126/10597	1.00 (reference)	206/5468	1.00 (reference)	121/5638	1.00 (reference)
HTLV-I (+)	45/2311	2.12 (0.92–4.89)	26/950	0.87 (0.43–1.78)	8/983	1.11 (0.42–2.93)
*TNF-*α 1031T/C						
CC & CT						
HTLV-I (−)	35/3766	1.00 (reference)	70/2059	1.00 (reference)	29/2120	1.00 (reference)
HTLV-I (+)	16/904	3.70 (0.82–16.7)	14/350	1.76 (0.62–5.03)	8/364	3.34 (0.93–12.0)
TT						
HTLV-I (−)	91/6801	1.00 (reference)	136/3384	1.00 (reference)	92/3493	1.00 (reference)
HTLV-I (+)	29/1386	1.90 (0.68–5.36)	12/581	0.46 (0.16–1.31)	0.2/599	0.33 (0.04–2.52)
*p* for interaction		0.122		0.176		0.020
*IL-10* 819T/C						
CC & CT						
HTLV-I (−)	94/6874	1.00 (reference)	132/3694	1.00 (reference)	102/3754	1.00 (reference)
HTLV-I (+)	38/1443	2.31 (0.87–6.15)	19/652	1.00 (0.41–2.41)	4/680	0.59 (0.13–2.55)
TT						
HTLV-I (−)	32/3709	1.00 (reference)	74/1764	1.00 (reference)	19/1874	1.00 (reference)
HTLV-I (+)	7/847	1.47 (0.28–7.68)	7/279	0.80 (0.23–2.77)	4/283	3.38 (0.77–14.9)
*p* for interaction		0.090		0.937		0.426
*NF-*κ*B1* 94ATTG ins/del						
ins/ins						
HTLV-I (−)	38/4554	1.00 (reference)	100/2321	1.00 (reference)	60/2412	1.00 (reference)
HTLV-I (+)	15/849	2.65 (0.63–11.1)	8/314	0.71 (0.25–2.05)	1.4/331	0.33 (0.04–2.58)
ins/del & del/del						
HTLV-I (−)	88/6019	1.00 (reference)	106/3132	1.00 (reference)	61/3211	1.00 (reference)
HTLV-I (+)	30/1441	1.86 (0.65–5.30)	18/617	1.03 (0.39–2.77)	7/632	2.55 (0.74–8.58)
*p* for interaction		0.238		0.904		0.155

CI, confidence interval; E, events; HR, hazard ratio; NE, non-events.

a Among the subjects in Amami and the mainland regions.

b Among the subjects in Amami region.

c Myocardial infaction, aortic aneurysm, and stroke.

d Adjusted for smoking habit, drinking habit, meat intake, hypertension, dyslipidemia, glucose intolerance, family history of CHD and stroke, overweight, and exercise habit.

We also compared the HR for total death with HTLV-I by region to evaluate its geographical variation. An increased HR was observed in the Amami island region in univariate analysis (HR 2.93; 95% CI, 1.17–7.37) and after adjustment for additional cancer and atherosclerosis related factors (HR 3.01; 95% CI, 1.18–7.70), but evaluation was difficult in the mainland due to small number of person-years (Table 4). The increased HR in the Amami island region was still statistically significant after additional adjustment for the three gene polymorphisms (HR 3.03; 95% CI, 1.18–7.77).

**Table 4:** Hazard ratios for total deaths with HTLV-I seropositivity according to regions in the sub-cohort study subjects.

	Total deaths			
	Person-years (E/NE)	HR^a^ (95% CI)	HR^b^ (95% CI)	HR^c^ (95% CI)
Amami islands				
HTLV-I (−)	110/7781	1.00 (reference)	1.00 (reference)	1.00 (reference)
HTLV-I (+)	42/1656	2.93 (1.17–7.37)	3.01 (1.18–7.70)	3.03 (1.18–7.77)
Mainland				
HTLV-I (−)	16/2816	1.00 (reference)	1.00 (reference)	1.00 (reference)
HTLV-I (+)	3/655	0.66 (0.08–5.36)	0.61 (0.07–5.07)	0.65 (0.08–5.35)

CI, confidence interval; E, events; HR, hazard ratio; NE, non-events.

a Univariate analysis.

b Adjusted for smoking habit, drinking habit, meat intake, hypertension, dyslipidemia, glucose intolerance, family history of CHD and stroke, overweight, and exercise habit.

c Adjusted for smoking habit, drinking habit, meat intake, hypertension, dyslipidemia, glucose intolerance, family history of CHD and stroke, overweight, exercise habit, and gene polymorphisms in *TNF-α* 1031T/C, *IL-10* 819T/C and *NF-*κ*B1* 94ATTG ins/del.

## Discussion

In the present matched cohort study, we observed a positive association between HTLV-I infection and the risk of total death in the Amami island region, which is in accordance with previous reports of increased risk of mortality for all causes other than ATLL and non-neoplastic diseases.^8^, ^9^, ^10^ Their association is still controversial, since several studies have not reported an increased risk.^40^, ^41^ However, one of these studies with no positive results may have had selection bias resulting from the healthy donor effect, and the other had limited statistical power due to small number of subjects and short follow-up period. The present association among combined subjects from the mainland and Amami island regions was not significant, though the statistical power was relatively low in the mainland. The present baseline survey in 2005–2012 was conducted 20 years after the previous surveys in 1985–1987 among atomic-bomb survivors in Nagasaki, aged 30–99 years,^8^ and in 1985–1992 among island residents in Nagasaki, aged 40–69 years.^9^ The present seropositive rates of 5.6% for men and 7.0% for women were much lower than the previous ones of 8.2% for men and 9.0% for women among atomic-bomb survivors and 22.9% for men and 26.2% for women among island residents. The present rates were also lower than rates of 12.5% for men and 15.1% for women reported in the same Kagoshima region in 1980–1984, after adjustment for age to the present subjects (personal calculation).^42^ A chronological decline in HTLV-I prevalence has been observed in the Kyushu district, including in the current study region and among the younger generation over the past two decades.^43^ Current HTLV-I prevalence comprises both the accumulation of three major transmission routes (breast feeding, blood transfusion, and sexual transmission)^1^ and the birth cohort effect on chronological change of each transmission route. Furthermore, the HTLV-I transmission route and timing during childhood and adulthood may play important roles in the association between HTLV-I infection and mortality and morbidity of diseases. There is no evidence on how chronological decline in HTLV-I prevalence influences this association. As the preventive intervention for HTLV-I transmission from mother-to-child and blood transfusion started at the end of 1980,^44^, ^45^ further study is expected in the future.

Geographical variation in SNP distribution among Japanese has been reported, and most Japanese individuals fell into two main clusters, Hondo from the main islands in Japan and Ryukyu from Okinawa.^46^ Another study showed genetic differentiation between the mainland population and Amami population using SNP distribution of J-MICC study subjects.^47^ The MAF of two gene polymorphisms was also different between Amami and mainland regions. Therefore, we adjusted for region to estimate the HRs for the interaction. Furthermore, the HRs for total death by Amami and mainland regions were estimated, after adjusted for inflammatory gene polymorphisms, and this adjustment did not alter the results.

The associations between inflammatory gene polymorphisms and the development of ATLL^23^ and HAM/TSP has been reported.^24^, ^25^, ^26^, ^27^ Interestingly, these polymorphisms were also associated with the development of not only several sites of cancer,^28^, ^29^, ^30^, ^31^, ^32^, ^33^ but also atherosclerosis-related diseases.^34^, ^35^, ^36^, ^37^ TNF-*α*, encoded by the *TNF*-α gene, is a cytokine responsible for resistance to infection and cancers and exerts major proinflammatory action by stimulating adhesion molecules and chemokine expression.^48^ Higher levels of the cytokine were observed not only in HAM/TSP cases,^49^ but also in asymptomatic and oligosymptomatic HTLV-I carriers.^50^ The present promoter-gene polymorphism *TNF*-α 1031T/C is functional in increasing gene transcription and cytokine levels.^34^ However, the association between the *TNF*-α 1031T/C polymorphism and cancer risk is controversial. Allele C was associated with an increased risk of gastric cancer^28^ and with a lower risk of squamous cell carcinomas of the nonoropharynx recurrence.^29^ TNF-*α* is involved in lipid metabolism, coagulation, insulin resistance, and endothelial function, while *TNF*-α 1031 allele C was associated with CHD risk and increased dyslipidemia.^34^ The present study observed the association between the *TNF*-α 1031T/C polymorphism and HTLV-I with an increased risk of atherosclerosis-related disease incidence. The HRs for atherosclerosis-related disease incidence with HTLV-I in CC and CT genotypes were consistently higher than those in the TT genotype, including in the case of total death and cancer incidence, although the HRs were not statistically significant.

IL-10 is a regulatory cytokine responsible for counterbalancing the inflammatory effects of pro-inflammatory cytokines, and its levels are decreased in HTLV-I-associated inflammatory diseases.^50^ The CC genotype of the *IL-10* 595A/C polymorphism showed a higher prevalence in HAM/TSP patients with higher HTLV-I provirus load.^26^ The C allele of the present *IL-10* 819T/C polymorphism was associated with increased risk for gastric cancer.^31^ Furthermore, the C allele was correlated to lower values of carotid IMT.^36^ Despite the given evidence, no significant interaction was observed between the present *IL-10* 819T/C polymorphism and HTLV-I for the risk of mortality and incidence with HTLV-I.

NF-*κ*B1 is an important transcription factor modulated by Tax, which plays a critical role in HTLV-I infection as a modulator of growth factors, cytokines, their receptors, and proto-oncogenes.^14^ A common deletion-allele in the promoter region of *NF-*κ*B*1 results in lower protein levels of the NF-κB p50 subunit. *NF-*κ*B1* 94ATTG ins/del polymorphism was associated with risk of cancer^32^, ^33^ and CHD.^37^ However, the present study did not find an interaction between *NF-*κ*B1* 94ATTG ins/del polymorphism and HTLV-I for the risk of total death or incidence of cancer and atherosclerosis-related diseases.

Several limitations of this study should be considered. First, the intermediate markers of HTLV-I associated diseases, such as anti-HTLV-I antibody titers, provirus load, or anti-Tax protein antibody, were not included. However, the present study used inflammatory gene polymorphisms to identify susceptible HTLV-I carriers, while a previous study reported a significant association between provirus load and inflammatory gene polymorphism.^26^ Second, an incidence survey was not conducted for mainland cohort subjects due to a shorter follow-up period, and the risk for incidence cases was estimated only among Amami subjects, with a survey coverage rate of 81.3%. The observed HRs for incidence cases may be slightly over-estimated due to misclassification because the positive predictive value for the diagnosis confirmation was relatively high but less than 100%. Third, the number of endpoints was relatively small, so we were unable to perform a sub-analysis for cause of deaths, type of cancer, and selected atherosclerosis-related diseases. As the follow-up is still under way, further analysis is expected in the future. Fourth, the number of analyzed inflammatory gene polymorphisms was limited. However, the selected gene polymorphisms play important functional roles in both HTLV-I infection status and the development of cancer and atherosclerosis-related diseases. Fifth, the genotype frequency of the *NF-*κ*B1* 94ATTG ins/del polymorphism was not in the HWE. The HWE deviation has two possible explanations: genotyping error or population stratification. Since its MAF (0.364) was not different between Japanese subjects overall and Japanese subjects from the Amami region (0.357),^33^ the probability of a genotyping error is low. The study subjects were selected from the general population of common Japanese ethnicity, and to our knowledge, no information has been obtained on the migration of a large-sized population with different MAF in the study regions.

In conclusion, the present study found an interaction between the *TNF*-α 1031T/C polymorphism and HTLV-I for the risk of atherosclerosis-related disease incidence, but not for the risk of total death and cancer incidence. Further follow-up study is warranted to understand the protective factors against developing diseases among susceptible HTLV-I carriers.

## Conflicts of interest

None declared.
